# Difluoromethylornithine Is a Novel Inhibitor of *Helicobacter pylori* Growth, CagA Translocation, and Interleukin-8 Induction

**DOI:** 10.1371/journal.pone.0017510

**Published:** 2011-02-28

**Authors:** Daniel P. Barry, Mohammad Asim, David A. Leiman, Thibaut de Sablet, Kshipra Singh, Robert A. Casero, Rupesh Chaturvedi, Keith T. Wilson

**Affiliations:** 1 Division of Gastroenterology, Department of Medicine, Vanderbilt University Medical Center, Nashville, Tennessee, United States of America; 2 Department of Cancer Biology, Vanderbilt University Medical Center, Nashville, Tennessee, United States of America; 3 Veterans Affairs Tennessee Valley Healthcare System, Nashville, Tennessee, United States of America; 4 Department of Oncology, Sidney Kimmel Comprehensive Cancer Center, Johns Hopkins University School of Medicine, Baltimore, Maryland, United States of America; Indian Institute of Science, India

## Abstract

*Helicobacter pylori* infects half the world's population, and carriage is lifelong without antibiotic therapy. Current regimens prescribed to prevent infection-associated diseases such as gastroduodenal ulcers and gastric cancer can be thwarted by antibiotic resistance. We reported that administration of 1% d,l-α-difluoromethylornithine (DFMO) to mice infected with *H. pylori* reduces gastritis and colonization, which we attributed to enhanced host immune response due to inhibition of macrophage ornithine decarboxylase (ODC), the rate-limiting enzyme in polyamine biosynthesis. Although no ODC has been identified in any *H. pylori* genome, we sought to determine if DFMO has direct effects on the bacterium. We found that DFMO significantly reduced the growth rate of *H. pylori* in a polyamine-independent manner. Two other Gram-negative pathogens possessing ODC, *Escherichia coli* and *Citrobacter rodentium*, were resistant to the DFMO effect. The effect of DFMO on *H. pylori* required continuous exposure to the drug and was reversible when removed, with recovery of growth rate *in vitro* and the ability to colonize mice. *H. pylori* exposed to DFMO were significantly shorter in length than those untreated and they contained greater internal levels of ATP, suggesting severe effects on bacterial metabolism. DFMO inhibited expression of the *H. pylori* virulence factor cytotoxin associated gene A, and its translocation and phosphorylation in gastric epithelial cells, which was associated with a reduction in interleukin-8 expression. These findings suggest that DFMO has effects on *H. pylori* that may contribute to its effectiveness in reducing gastritis and colonization and may be a useful addition to anti-*H. pylori* therapies.

## Introduction

*Helicobacter pylori* is a Gram-negative bacterial pathogen that selectively colonizes the human stomach. It infects more than 50% of the world's population and is the causative agent of disorders that arise from the resulting chronic inflammation, ranging from dyspepsia and gastritis to gastric and duodenal ulcers [1]. It is also strongly associated with development of gastric cancer, the second most common cause of cancer death [2]. *H. pylori* infection usually occurs early in life and although there is an immune response elicited by the organism, it is insufficient to eliminate the bacteria. Infection therefore generally persists for the lifetime of the host, though cure is possible with antimicrobial therapy. The current standard of care prescribed to eliminate *H. pylori*–concurrent administration of a proton pump inhibitor and two antibiotics–is not effective for all patients, and in some regions eradication rates can be as poor as 50% [3], [4], [5]. As antibiotic resistance is continually rising, novel chemotherapeutic agents are of interest.

We recently reported that chronic administration of 1% (w/v) d,l-α-difluoromethylornithine (DFMO) in the drinking water to mice infected with *H. pylori* SS1 led to a reduction in both the level of colonization and the severity of gastritis [6]. DFMO is an analogue of the amino acid ornithine, which in eukaryotes and many bacteria is a substrate of ornithine decarboxylase (ODC), the enzyme responsible for catalyzing the conversion of the amino acid into the polyamine putrescine, which can then be converted to the polyamines spermidine and spermine by specific synthases. As such, DFMO serves as an irreversible inhibitor of ODC [7]. Polyamines are polycationic molecules present in all living organisms that are essential for the synthesis of macromolecules (e.g. DNA, RNA and protein) [8], [9], [10]. The pathways of polyamine biosynthesis are well-understood for eukaryotes and for the model bacterial species *Escherichia coli* [11], but the full mechanisms of polyamine production in *H. pylori* have not been described and no sequenced strain possesses an ODC [12], [13], [14], [15]. However, because of the clinical efficacy that we had observed *in vivo*, we investigated whether DFMO has any direct effect on *H. pylori* that could account, at least in part, for its effectiveness in reducing inflammation and bacterial load in infected mice.

We now report that growth of *H. pylori* in broth cultures supplemented with 1% DFMO was substantially inhibited and that the generation time was nearly doubled, but that the effects of DFMO were not associated with alterations in polyamine levels and could not be reversed with polyamine supplementation. In addition, exposure to DFMO induced a morphological change in the bacteria, such that they appeared closer to a coccoid form, and also caused elevated intracellular levels of ATP. In gastric epithelial cells, the prototypical inflammatory response to *H. pylori* infection is the induction of the neutrophil chemoattractant IL-8 [16], [17], which has been associated with injection of the product of the *H. pylori* gene cytotoxin associated gene (*cag*) *A*, namely CagA, into these cells by a type IV secretion system (T4SS), followed by its phosphorylation [18]. We now demonstrate that *H. pylori* exposed to DFMO for only 12 h showed a marked reduction of *H. pylori* CagA protein expression as well as less translocation of CagA and decreased phospho-CagA within human gastric epithelial cells. Moreover, DFMO-treated bacteria exhibited a reduced ability to induce mRNA expression and secretion of IL-8 in these cells. These studies indicate that direct effects of DFMO on *H. pylori* may be important for the drug's effectiveness *in vivo* and that polyamine-independent effects of DFMO mediate its effects on the bacterium.

## Materials and Methods

### Bacterial cultures

*H. pylori* strains SS1 [19] and 60190 [20] were maintained by passage on plates of tryptic soy agar containing 5% sheep blood, as described [21]. Prior to experiments, bacteria were grown overnight in Brucella broth supplemented with 10% FBS in upright 25-cm^2^ tissue culture flasks at 37°C with 5% CO_2_ at 120 rpm. *E. coli* and *Citrobacter rodentium* were maintained on LB plates and starter cultures were grown overnight in LB broth at 37°C at 200 rpm, or with no agitation, respectively [22], [23].

### Bacterial growth curves

Overnight starter cultures of *H. pylori* were used to inoculate new Brucella broth cultures at 0.1 OD_600_. Bacterial growth was monitored by optical density for 24 h. To quantify viable bacteria, culture samples were diluted and plated and colonies were counted once visible. Some cultures were supplemented with DFMO, ornithine, putrescine, or spermidine alone or in combination and differences in growth were measured. As addition of DFMO slightly acidified the media, all were adjusted with sodium hydroxide to a pH of ∼6.9 and filter sterilized prior to inoculation with bacteria. We also measured the growth of *H. pylori* in F12 medium supplemented with 10% FBS and 2 mM glutamine. The effect of DFMO on *C. rodentium* and *E. coli* was determined in LB broth cultures begun at 0.05 OD_600_ and bacterial growth was monitored by optical density.

### Ornithine decarboxylase activity assay

To determine if *H. pylori* possessed any unrecognized ODC activity, bacteria were grown for 6 h in Brucella broth with or without 1% DFMO supplementation and then lysed by sonication in buffer consisting of 50 mM Tris (pH 7.5), 2.5 mM dithiothreitol, 1 mM EDTA and 40 µM pyridoxal 5′-phosphate. ODC activity was measured by radiometric analysis of ^14^CO_2_ liberated from l-[^14^C]ornithine, as described previously [24]. *E. coli*, which are known to possess an ODC, were grown for 6 h in LB and treated in the same way to serve as a positive control.

### Polyamine measurement

*H. pylori* were grown in broth with or without 1% DFMO supplementation. Samples of growth medium and bacteria were taken over the 12 h time course. Broth samples were centrifuged to remove bacteria and frozen until analysis. Equal numbers of bacteria (1×10^7^ estimated from OD_600_) were washed multiple times in phosphate buffered saline and then resuspended and frozen in lysis buffer (2 mM DTT, 2 mM EDTA, 10% glycerol, 1% Triton X-100, 25 mM Trizma-HCl, pH 8.0). Polyamine levels in growth media and bacterial lysates were determined by precolumn dansylation reverse phase high performance liquid chromatography as reported previously [25], [26].

### Mouse infection

Male C57BL/6 mice aged 6–8 weeks were infected as described [6], [24], [27]. Briefly, *H. pylori* SS1 were grown for 12 h with or without 1% DFMO and 5×10^8^ bacteria were delivered intragastrically on days 0, 2 and 4. After one month, mice were sacrificed and colonization was determined by serial dilution and plating of gastric homogenates. After one week colonies were counted to determine bacterial load. This study was carried out following recommendations in the Guide for the Care and Use of Laboratory Animals of the National Institutes of Health. The protocol was approved by the Institutional Animal Care and Use Committee of Vanderbilt University (Protocol Number V/07/247).

### *H. pylori* morphological assessment

Experiments were performed to determine the permanence of the inhibitory properties of DFMO on *H. pylori*. Brucella broth cultures were started with or without DFMO. After 6 or 12 h of growth, the cultures were collected and pelleted by centrifugation. The bacteria were then resuspended in media with or without DFMO such that four different growth conditions resulted. Growth was monitored by optical density. In addition, bacterial morphology was determined using Gram stain to monitor changes induced by exposure to DFMO. To more precisely quantify size variations, transmission electron microscopy (TEM) was used. Culture aliquots were removed, pelleted by centrifugation and washed in PBS. The bacteria were then fixed with 2.5% gluteraldehye and then washed in 0.1 M sodium cacodylate buffer. The samples were postfixed in 1% aqueous osmium tetroxide, dehydrated through a graded series of ethanols to 100% ethanol, and then embedded in Spurr Resin. Thin sections of the resin-embedded material were examined using a Philips CM-12 high-resolution transmission electron microscope and images were captured by a CCD camera at 11,500X magnification. The size of the bacteria was determined by analyzing the images with NIH ImageJ (version 1.41o) to measure the area of identified *H. pylori*. Briefly, the threshold command was used to separate bacteria from the background and the two-dimensional area of each cell was calculated in µm^2^ [28].

### ATP assay

The CellTiter-Glo assay (Promega) was used to determine the relative amount of ATP present in bacteria over the course of growth as an indicator of metabolic activity. *H. pylori* SS1 were grown in Brucella broth with or without DFMO and then analyzed. Briefly, a volume of bacteria was removed, pelleted, and washed multiple times in phosphate buffered saline. The bacteria were then resuspended and frozen in lysis buffer (2 mM DTT, 2 mM EDTA, 10% glycerol, 1% Triton X-100, 25 mM Trizma-HCl, pH 8.0) and luminescence proportional to ATP level was assayed according to the kit's instructions. Levels per µL of culture were calculated, as were levels per 10^6^ bacteria using the measured optical density at each time point.

### Gene expression in epithelial cells

IL-8 mRNA was measured in AGS epithelial cells exposed to *H. pylori* 60190. AGS cells were grown in F12 medium supplemented with 10% FBS and 2 mM glutamine. *H. pylori* from a starter culture were grown for 12 h with or without exposure to DFMO at which time the bacteria were collected and washed with PBS. In 6-well plates, 5×10^5^ AGS cells per well were infected with *H. pylori* at a multiplicity of infection (MOI) of 200 bacteria per cell or were uninfected for a negative control. Total RNA was isolated 4 h after exposure, using TriZOL (Invitrogen), and cDNA was synthesized from 1 µg RNA, as described [29]. Real-time PCR was performed in a LightCycler 480 (Roche). Relative gene expression was calculated from second derivative maximum values using β-actin as a reference gene. IL-8 primers were 5′-TAGCAAAATTGAGGCCAAGG-3′ and 5′-AAACCAAGGCACAGTGGAAC-3′. β-actin primers were as described previously [24].

### IL-8 protein expression in epithelial cells

AGS cells were grown as described above and were infected with *H. pylori* 60190 at an MOI of 100. Six h after exposure, cell supernatants were collected. IL-8 protein concentrations were determined using a DuoSet human IL-8 ELISA kit (R&D Systems) according to the manufacturer's instructions.

### Adherence of *H. pylori* to gastric epithelial cells

AGS cells and *H. pylori* 60190 were grown as described above and cocultured at an MOI of 100 for 2 h with mild agitation every 20 minutes. Unbound bacteria were removed by washing three times with PBS. The cells were then incubated at 37°C for 15 minutes with PBS containing 0.1% saponin to free AGS-associated *H. pylori*. The bacteria were serially diluted and plated and colonies were counted after 5 days of growth [30].

### Protein expression in *H. pylori*

*H. pylori* 60190 was grown with or without 1% DFMO supplementation for 12 h. An isogenic 60190 *cagA^–^* mutant was used as a control [31]. Bacteria were concentrated by centrifugation, washed with PBS and lysed in buffer containing 150 mM NaCl, 1% NP40, and 50 mM Trizma-HCl (pH 8.0). 5 µg of protein were resolved by electrophoresis on a 4–20% Tris-HCl polyacrylamide gel (Bio-Rad) and transferred overnight onto a PVDF membrane, which was then blocked in 5% milk for 2 h. CagA was detected with 1∶2000 polyclonal anti-*H. pylori* CagA antibody (Austral Biologicals) followed by 1∶5000 horseradish peroxidase-conjugated anti-rabbit secondary antibody (Jackson ImmunoResearch). Urease was detected with 1∶10,000 polyclonal anti-*H. pylori* UreB antibody [32] followed by 1∶5000 horseradish peroxidase-conjugated anti-rabbit secondary antibody (Jackson ImmunoResearch). Signals were detected using SuperSignal West Pico peroxidase substrate (Thermo Scientific).

### mRNA expression in *H. pylori*

*H. pylori* 60190 was grown for 6 h or 12 h. Total RNA was extracted from 10^8^ bacteria using TriZOL, and cDNA was synthesized from 1 µg RNA. Real-time PCR was performed in a LightCycler 480 (Roche). Relative gene expression was calculated from second derivative maximum values using 16S rRNA as a reference gene. *cagA* primers were 5′-ACCAACAAGGTAACAATGTGGC-3′ and 5′-TCGTTGTGAGCCTGTGAGTTGGT-3′. *cagE* primers were 5′-CAATGGGTGGGGAGTATGTC-3′ and 5′-TGCTCCATTGTTGCATTTGT-3′. *cagM* primers were 5′-GGTTGCGTTTGGAGTTTTGTCGGC-3′ and 5′-AGCGTCTTCTTTTGCGGCCACT-3′. 16S primers were 5′-CAGCTCGTGTCGTGAGATGT-3′ and 5′-CGTAAGGGCCATGATGACTT-3′.

### Determination of *cagA* transcript stability

*H. pylori* 60190 was grown with or without 1% DFMO supplementation for 6 h at which time 100 µg/mL rifampicin was added to halt RNA transcription. At 15 min intervals aliquots of *H. pylori* containing 10^8^ bacteria were removed, washed twice in PBS and resuspended in TriZOL. Transcript levels of *cagA* were determined by real-time PCR as described above and normalized to stable 16S rRNA levels [33]. Transcript half-lives were determined by fitting exponential decay curves to the data.

### Detection of CagA translocation

2.5×10^6^ AGS cells were plated in 60-mm^2^ tissue culture dishes and were infected for 6 or 12 h at an MOI of 100 with *H. pylori* 60190 grown for 12 h, with or without DFMO. The cells were removed by trypsinization and lysates were prepared by three 10-second pulses of sonication at 40 W (Ultrasonic Processor GE 130PB, Hielscher) in buffer composed of 50 mM Tris-HCl (pH 8.0), 150 mM NaCl, 1% (v/v) NP-40, and 0.1% (w/v) sodium dodecyl sulfate. 25 µg of protein were resolved by electrophoresis on 10% Tris-HCl polyacrylamide gels (Bio-Rad) and transferred overnight onto PVDF, which was then blocked in 3% BSA for 1 h. Phosphorylated proteins were detected with 1∶300 diluted anti-phosphotyrosine antibody (Santa Cruz Biotechnology) followed by 1∶3000 horseradish peroxidase-conjugated anti-mouse secondary antibody (Jackson ImmunoResearch). Signals were detected using SuperSignal West Pico peroxidase substrate. After antibody stripping (Restore Western blot stripping buffer, Thermo Scientific) and reblocking in 5% milk, blots were probed with 1∶2000 polyclonal anti-*H. pylori* CagA antibody (Austral Biologicals), followed by 1∶3000 horseradish peroxidase-conjugated anti-rabbit secondary antibody (Jackson ImmunoResearch). As a loading control, β-actin levels were detected using 1∶20,000 anti-β-actin antibody (Sigma) followed by 1∶10,000 anti-mouse secondary antibody (Jackson ImmunoResearch).

### Urease activity assay

We used a modification of the method of Nagata, et al. to measure urease activity in bacteria [34]. Briefly, *H. pylori* SS1 or 60190 were grown in Brucella broth with or without 1% DFMO. After 3 or 6 h aliquots of bacteria were removed, washed in PBS and rapidly frozen in liquid nitrogen. Bacteria (1×10^6^) were incubated in a detection solution consisting of 0.0001% (w/v) phenol red and 100 mM urea in PBS and OD_550_ readings were taken over 60 min. Activity was determined from the slope of a best-fit line on a plot of OD_550_ versus time and was normalized to values obtained for control cultures.

### Statistical analysis

Data are expressed as means ± standard error. Student's *t* test was used for pairwise comparisons. Data from more than two groups was analyzed by ANOVA followed by the Newman-Keuls post hoc multiple comparisons test. The Kruskal-Wallis test followed by the Dunn's multiple comparisons test was used for the bacterial size data.

## Results

### Growth of *H. pylori* is inhibited by DFMO

**Figure 1:**
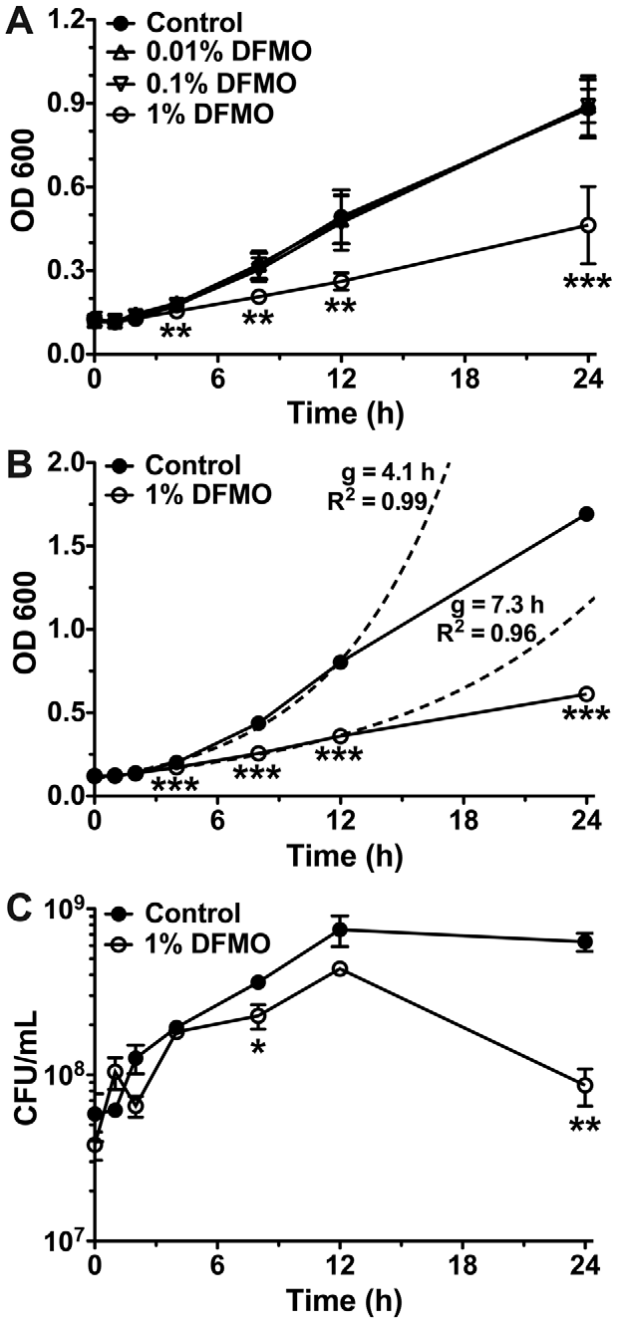
DFMO suppresses the growth of *H. pylori*. Brucella broth cultures, some containing DFMO, were inoculated with *H. pylori* SS1 at an OD_600_ of ∼0.1 and growth was monitored for 24 h. (A) Bacteria were grown in control broth or broth supplemented with 0.01%, 0.1%, or 1% (w/v) DFMO and growth was monitored by measuring OD_600_ at the indicated time points. Solid lines depict the growth curve obtained for each treatment and error bars represent the standard error (*n = *3). (B) *H. pylori* were grown and monitored as described for panel *A* in control broth or broth with 1% DFMO. Solid lines are experimentally obtained growth curves, with standard errors (*n = *9), while the dashed lines indicate the calculated exponential regression curves using the first 12 h of data. The generation time (g) and goodness of fit (R^2^) are indicated for each curve. (C) Bacteria were cultured as in panel B, and at the indicated time points samples were collected, diluted and plated on solid medium. Colonies were counted once visible to calculate concentrations of viable bacteria. Solid lines depict the growth curve obtained for each treatment and error bars represent the standard errors (*n* = 3). For all graphs, *, *p<*0.05; **, *p<*0.01; ***, *p<*0.001 *versus* the control condition.

We recently reported that in mice infected with the mouse-adapted strain *H. pylori* SS1, administration of 1% DFMO suppressed both bacterial colonization and gastric inflammation [6]. Notably, this is the same dose of DFMO that has been shown to be efficacious in mouse models of colon tumorigenesis [35]. Although no sequenced *H. pylori* genome has been found to contain an ODC, we investigated whether DFMO had any direct effect on the bacterium. *H. pylori* are primarily found in the surface mucous layer of the stomach, and we began with the assumption that in our *in vivo* mouse model the 1% DFMO in the drinking water would reach the *H. pylori* bacteria at this concentration, and the DFMO is replenished continuously by ongoing intake of this agent in the drinking water. Therefore, we initiated our studies with 1% DFMO (55 mM) in liquid culture media. Flasks of broth, some supplemented with different percentages of DFMO, were inoculated with *H. pylori* SS1 at an OD_600_ of 0.1 and growth was monitored by optical density for 24 h (Figure 1A). Growth of *H. pylori* in broth containing 1% DFMO was restricted beginning 4 h post-inoculation, while lower concentrations had no effect. When we repeated these growth curves to quantify the inhibitory effects of the chemical, we observed that *H. pylori* SS1 grown in standard broth had a generation time of 4.1 h during the exponential growth phase from 0–12 h (Figure 1B). In contrast, 1% DFMO significantly increased the generation time to 7.3 h (*p<*0.001), an increase of 78% (Figure 1B). We also monitored growth of *H. pylori* by diluting and plating the bacteria at time points from 0 to 24 h. At 8 h there were 38±7% fewer viable, culturable bacteria and at 24 h there was an 86±4% decrease (Figure 1C). These findings indicated that there was a direct effect of DFMO on *H. pylori* that was observable as a decrease in growth rate.

### The mechanism of growth inhibition does not involve polyamines

**Figure 2:**
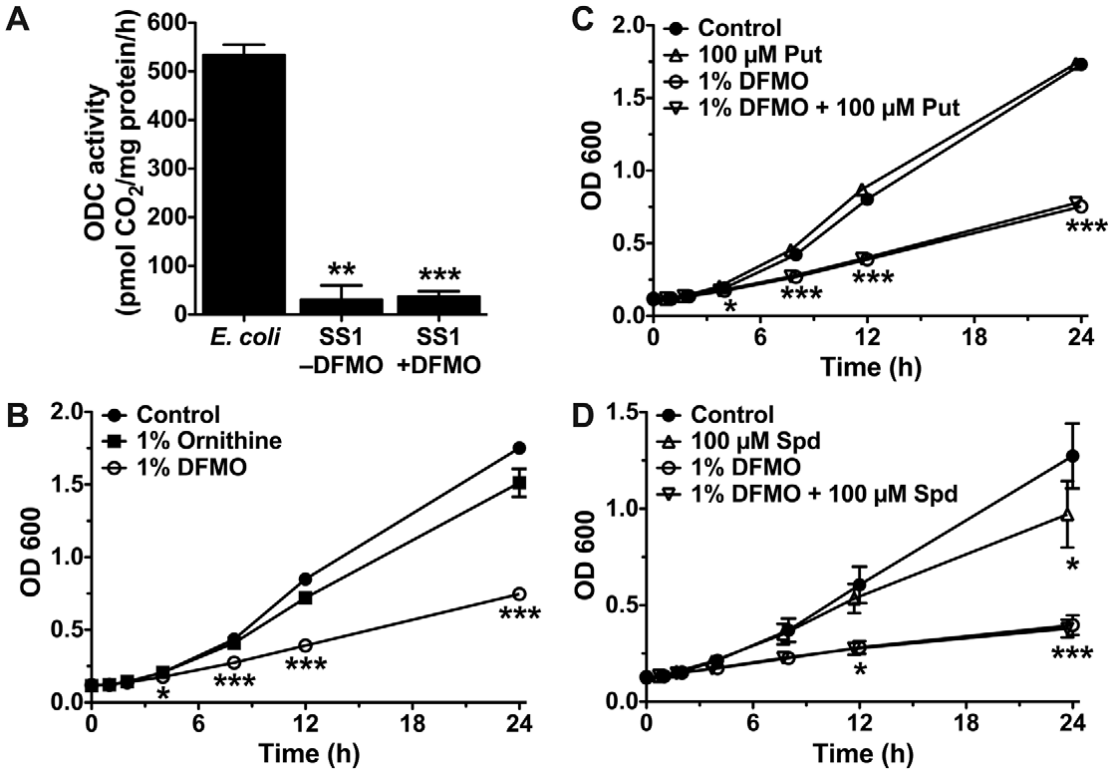
Polyamine pathways are not involved in the growth-suppressive effect of DFMO. (A) Bacteria were grown for 6 h with or without 1% DFMO, then washed and lysed by sonication. Lysates were incubated with ^14^C-labeled ornithine and ODC enzyme activity was determined by quantification of released ^14^CO_2_, normalized to total protein level and time, after subtracting lysate-free background values. **, *p<*0.01; ***, *p<*0.001 *versus* activity in *E. coli*. (B), (C), (D) Cultures were supplemented as labeled and grown and monitored by OD_600_ as described for Figure 1 (*n = *3). For panels *B*, *C* and *D*, *, *p<*0.05; ***, *p<*0.001 *versus* the control condition. Put, putrescine; Spd, spermidine.

DFMO is an irreversible inhibitor of ODC, an enzyme in the polyamine pathway of many organisms. However, as no homologue of this enzyme has been identified by sequence analysis in any *H. pylori* genome, we suspected that an alternative mode of action was responsible for the effect of DFMO on these bacteria. Although *H. pylori* contains no recognized ODC, homologous enzymatic activity could still be present. We therefore measured ODC activity in lysates of bacteria cultured with or without 1% DFMO. In contrast to *E. coli* DH5α, which exhibited substantial ODC activity, decarboxylation of ornithine in *H. pylori* lysates was 93.8±2.3% lower (*p<*0.01) and was not affected by DFMO (Figure 2A). In addition, polyamine levels measured in *H. pylori* lysates and culture supernatants were unaffected by DFMO treatment (Figures S1 and S2), suggesting that the mechanism of action of DFMO does not involve alterations to polyamine metabolism.

We next investigated if the inhibitory effect of DFMO on *H. pylori* growth was shared by the amino acid ornithine, which has a similar chemical structure. To test this, we compared *H. pylori* SS1 grown in broth supplemented with 1% DFMO or an equimolar concentration of l-ornithine. The addition of l-ornithine had no effect on the growth of the bacteria (Figure 2B).

The culture medium used to grow the bacteria—Brucella broth plus 10% FBS—is a rich polyamine-containing medium and analysis by HPLC indicated that it contained 4.94 µM putrescine and 9.74 µM spermidine (Figure S1). We attempted to restore growth kinetics to control levels by supplementation with even higher levels of exogenous polyamines. We did not attempt spermine supplementation, as it has been reported that this polyamine is not present in *H. pylori* [36]. Attempts to counteract the inhibitory effect of DFMO by addition of either 100 µM putrescine (Figure 2C) or 100 µM spermidine (Figure 2D) had no effect, and spermidine alone actually had a modest inhibitory effect at 24 h. However, the reported absence of any identified polyamine transporters in this bacterium [11] could account for our observations.

### DFMO does not inhibit the growth of other Gram-negative bacteria

**Figure 3:**
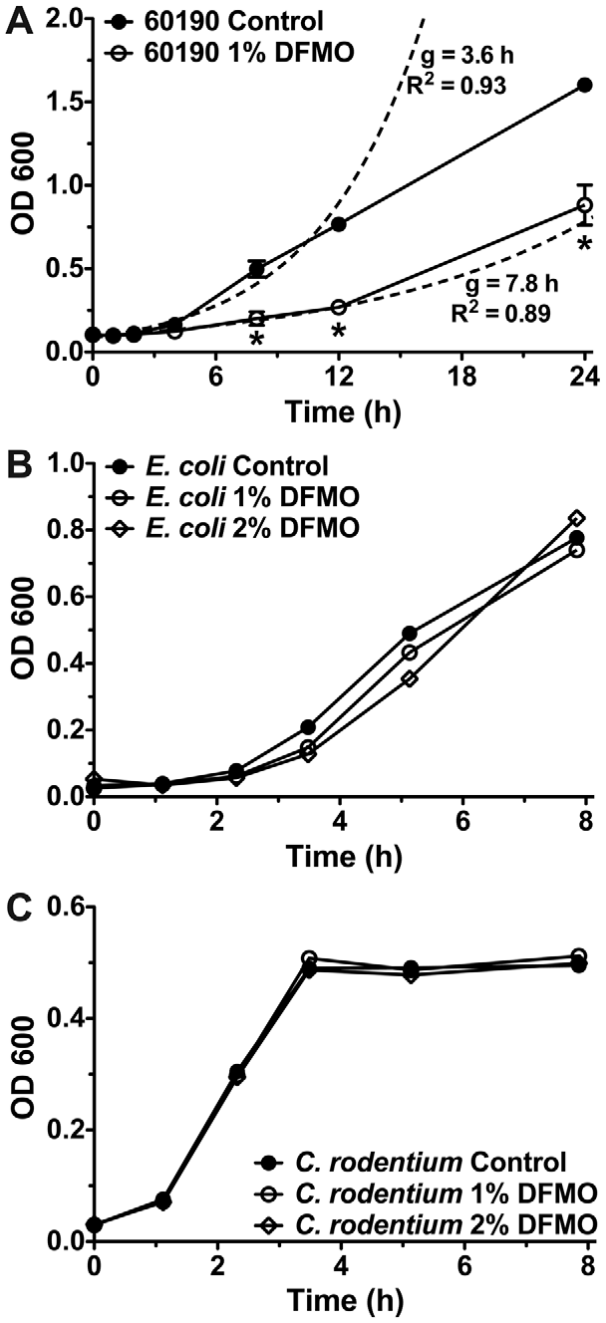
DFMO affects *H. pylori* 60190, but has no effect on two other Gram-negative bacteria. (A) Growth rates of *H. pylori* 60190 cultured in the absence or presence of 1% DFMO were compared as described in Figure 1B. Dashed lines indicate exponential curve regressions for each data set and are labeled with generation time and goodness of fit (*n = *3; *, *p<*0.05). (B) *E. coli* DH5α were grown in control broth or broth supplemented with 1% or 2% DFMO and growth was monitored for 8 h. (C) *C. rodentium* were grown in control broth or broth supplemented with 1% or 2% DFMO and growth was monitored for 8 h.

As our experiments to this point utilized a single strain of *H. pylori*, SS1, and the *H. pylori* species is known to exhibit an extreme degree of genetic diversity [37], we next investigated the growth inhibitory effect of DFMO on a second strain of *H. pylori*, 60190, that has been extensively used in laboratory studies [38], [39]. When we tested the effect of 1% DFMO on the growth of strain 60190 we observed a similar outcome, with generation time increasing from 3.6 h to 7.8 h (Figure 3A). We then explored whether the effect of DFMO was more universal and could be seen in other bacterial species. We selected *E. coli* and *C. rodentium*, which are Gram-negative bacteria of the same phylum as *H. pylori* (Proteobacteria). Neither of these species were affected by 1% DFMO, nor was there an effect when we doubled the concentration to 2% (Figures 3B and 3C). The fact that no inhibition of growth was observed using *E. coli* or *C. rodentium*, close relatives of *H. pylori* that do express ODC, suggests that the effect may be species-specific and is not simply due to toxicity, and that the mechanism is likely polyamine-independent.

### The effect of DFMO is not permanent

**Figure 4:**
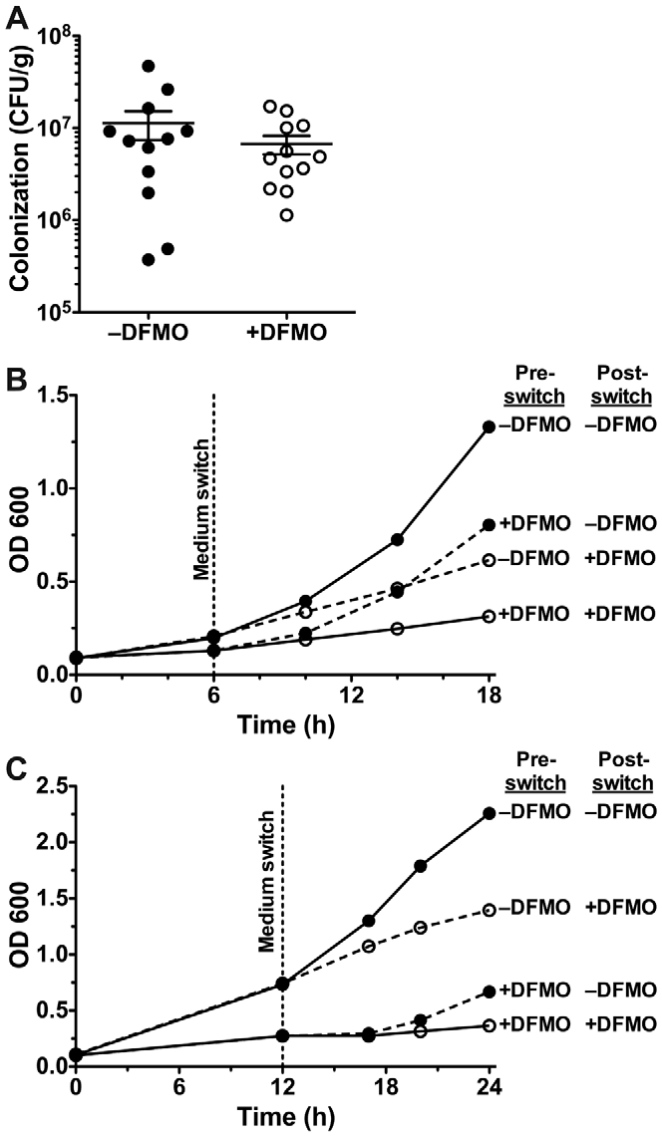
The effect of DFMO on *H. pylori* requires constant exposure. (A) Mice were infected with *H. pylori* SS1 grown for 12 h with or without 1% DFMO and colonization levels were determined after one month. Each point represents bacterial load in a single mouse, and mean and standard error are indicated (*n = *12 per group). (B) Growth curves were performed to determine the permanence of the effect of DFMO. Brucella broth cultures, with or without DFMO, were inoculated with *H. pylori*. After 6 h, the cultures were centrifuged and switched to new medium so that four conditions resulted: –DFMO → –DFMO, +DFMO → –DFMO, –DFMO → +DFMO, and +DFMO → +DFMO. Bacterial levels were monitored by OD_600_. Data depicted are from a single representative experiment; similar results were obtained in two others. (C) Conditions are the same as for panel B, except that medium switching occurred after 12 h of growth. Data are from a single representative experiment of three performed.

In our previous study, we reported that mice infected with *H. pylori* SS1 and then treated continuously with 1% DFMO for 4 months demonstrated reduced colonization and gastritis. Our current findings suggest that this effect could involve direct alteration of bacterial metabolism as well as the known inhibition of host cell ODC. In an attempt to separate these effects, we infected mice with *H. pylori* cultured for 12 h with or without 1% DFMO prior to inoculation, in the absence of *in vivo* treatment of the animals, and quantified bacterial colonization after 1 month by homogenizing a portion of the stomach and plating dilutions on solid medium. We found no difference in the level of colonization at this time point (Figure 4A), suggesting that the effect of DFMO required continuous exposure to the chemical and that in the absence of DFMO, *H. pylori* could return to its unaffected state. To further examine the permanence of these effects we carried out experiments in which bacterial cultures were begun in one medium—either with or without 1% DFMO—and then switched after 6 h of growth, a time point at which growth suppression and metabolic perturbation is already apparent. Figure 4B illustrates that cultures maintained in one medium (i.e. no DFMO, or continuous DFMO) exhibited growth patterns identical to those seen in earlier experiments. In addition, cultures begun in unadulterated medium and then switched to broth containing DFMO showed a nearly immediate reduction in growth rate. Importantly, the cultures initiated in broth containing 1% DFMO that were then switched to plain medium at 6 h were able to recover very quickly, so that by 12 h of additional growth they had attained the same growth rate as the cultures continuously grown without DFMO (generation time of 4.4 h vs. 4.5 h, *p = *0.363). This rapid resurgence suggested that rather than being killed by exposure to DFMO, the chemical instead had a bacteriostatic effect. When we repeated these experiments with switching delayed until 12 h had elapsed (Figure 4C) the results were similar, except that those *H. pylori* transferred from 1% DFMO to control medium exhibited a lesser ability to recover compared to bacteria switched after 6 h. After 12 h of additional growth, generation time in continuous control medium was 7.0 h while that in the cultures switched from DFMO to normal medium was only 9.2 h (*p* = 0.0502).

### DFMO alters cell morphology

**Figure 5:**
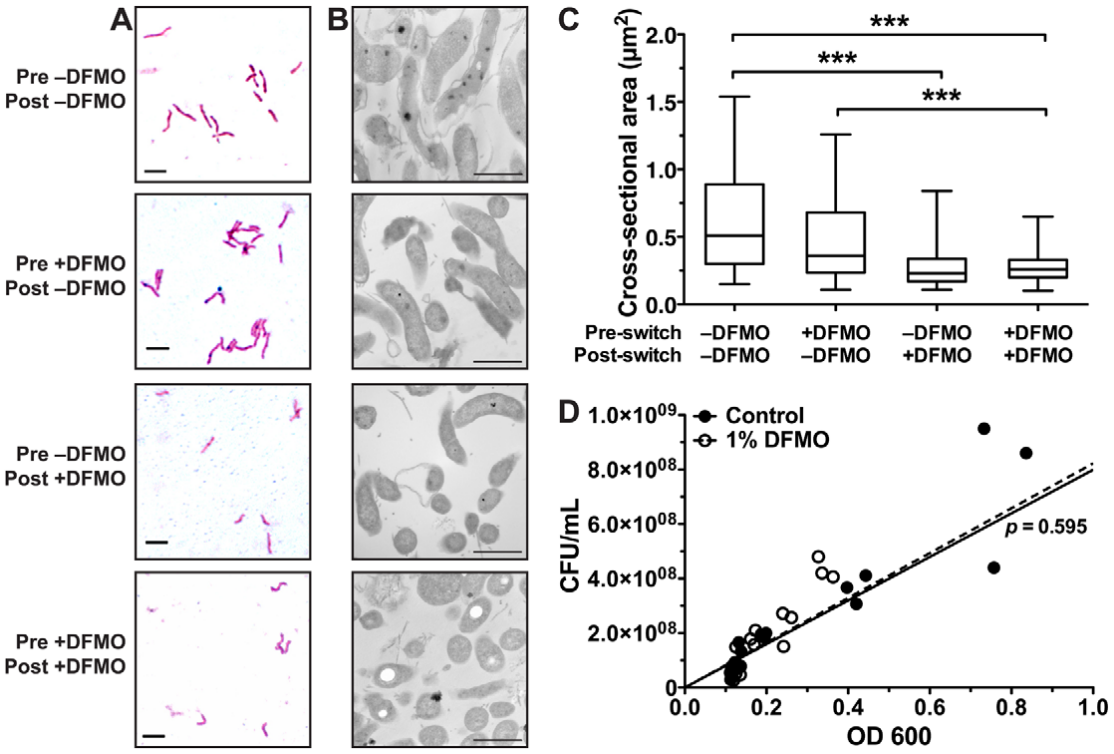
DFMO alters bacterial morphology. Bacterial samples were removed from cultures 6 h after the medium switch depicted in Figure 4B. Gram stains were performed and examined by light microscopy (A) and bacterial pellets were fixed, sectioned, and stained for TEM (B). Note that the labels for panel A also apply to panel B. Scale bars indicate 2 µm in panel *A* and 1 µm in panel B. (C) Stained bacteria were examined by TEM and cross-sectional areas were measured. For each treatment group the box identifies the median and 25^th^ and 75^th^ percentile values and the whiskers indicate the range of measurements (*n = *83–129 measurements per group). ***, *p<*0.001 between groups indicated. *D*, a plot of OD_600_ *versus* bacterial concentration in CFU/mL was created from the data points of the first 12 h of growth curves depicted in Figures 1B and 1C (*n = *9 per treatment). The lines illustrate the best-fit linear regression of each treatment group (control, solid line; 1% DFMO, dashed line) constrained to pass through the origin. The two lines did not significantly differ (*p = *0.595). The global best-fit line (not depicted, R^2^ = 0.80) indicated that an OD_600_ of 1 was equivalent to 8.11×10^8^ CFU/mL.

Due to the manner of bacterial replication, via binary fission, growth rate can be linked with bacterial size. We therefore determined if DFMO induced any alterations in bacterial morphology. To test this, we sampled bacteria from the 6 h switching experiment and performed Gram stains at 6 h after the medium switch. The bacteria from cultures containing 1% DFMO appeared to be smaller than bacteria from cultures without DFMO (Figure 5A). Due to the low resolution afforded by light microscopy, we also prepared samples of bacteria for transmission electron microscopy. Sections of bacterial pellets were examined (Figure 5B) and the sizes of individual bacteria were determined using NIH ImageJ software. As was suspected from the Gram stains, bacteria cultured in the presence of 1% DFMO were significantly smaller than those cultured in plain broth (*p<*0.001). We also observed that 6 h after removal of DFMO bacterial size had significantly increased (*p<*0.001), and that 6 h of culture with DFMO after switching was sufficient to significantly reduce bacterial size (*p<*0.001) (Figure 5C). These data indicate that *H. pylori* morphology is rapidly altered by exposure to DFMO, but that the effect disappears once the chemical is removed.

The discovery that bacterial morphology was altered by exposure to DFMO caused us to reexamine our investigations using optical density, as such measurements could be dependent on bacterial size and shape. To allay these concerns we plotted bacterial concentration (CFU/mL) *versus* OD_600_ for bacteria grown with or without 1% DFMO for up to 12 h (Figure 5D). When constrained to pass through the origin, linear regression analysis of the two data sets found no significant difference (*p = *0.595) and revealed that an OD_600_ of 1 was equivalent to 8.11×10^8^ CFU/mL. This finding indicates that the changes in bacterial size did not affect the estimations of bacterial concentration, and thus the growth curve data reported above required no reanalysis.

### DFMO affects bacterial metabolism

**Figure 6:**
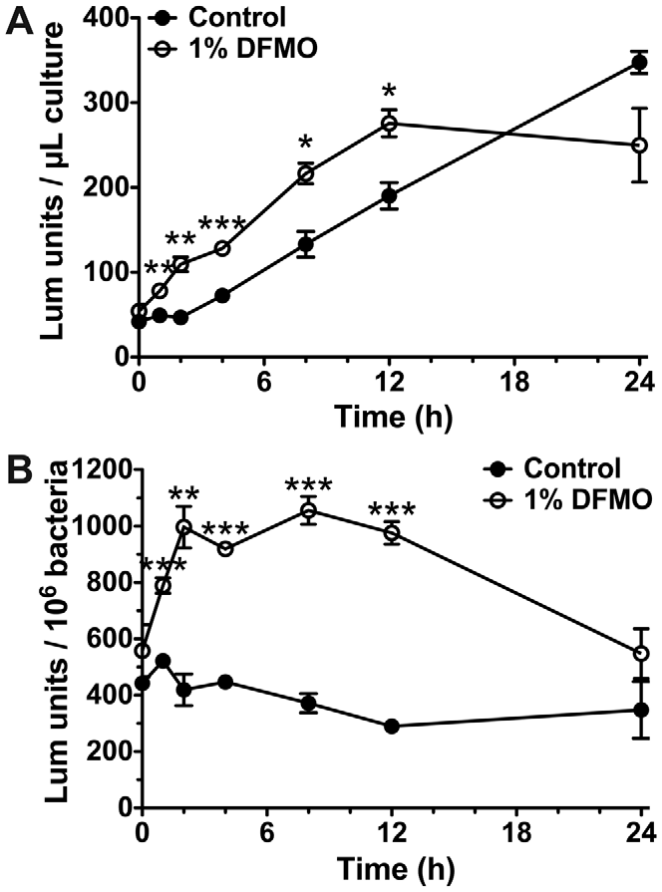
Metabolic activity is altered in *H. pylori* cultured with 1% DFMO. Cultures were grown for 24 h and bacterial samples were removed at the indicated time points. Relative ATP levels were measured in bacterial lysates using CellTiter-Glo, a luciferase-based assay kit. (A) Luminescence (Lum) units, proportional to ATP concentration, were calculated for equivolumetric samples. (B) Luminescence units per 10^6^ bacteria at each time point were calculated using the data from panel *A*, the observed OD_600_ of the culture, and the equation derived from Figure 5*D*. Each data point is the average of 3 experiments with the standard errors shown. For both graphs, *, *p<*0.05; **, *p*<0.01; ***, *p<*0.001 *versus* the control condition.

Monitoring bacterial growth by optical density has inherent limitations, as both live and dead cells can remain in suspension and contribute to an apparent increase in the number of growing bacteria. We attempted to circumvent this problem by quantifying viable cells by diluting and plating the culture on solid medium. Although we did begin to see a decrease in the number of culturable bacteria after 8 h of culture (Figure 1C), optical density levels diverged 4 h prior (Figure 1B) and significant die-off of *H. pylori* was not seen until 24 h of culture with 1% DFMO. Our inference was that rather than having a bactericidal effect, DFMO was altering cell metabolism, which led to a significant decrease in growth rate. To determine if this was the case, we utilized a luciferase-based assay to measure ATP levels in bacterial lysates. Unexpectedly, rather than inducing a decrease in ATP, DFMO had the opposite effect. When we analyzed equal volumes of cultures grown with or without 1% DFMO, we observed a significant increase in ATP levels beginning only 1 h after initiation (Figure 6A). ATP levels per microliter increased steadily over time for both cultures until the 24 h time point, when levels in DFMO-containing cultures leveled off. For control bacteria there was obvious parallelism between the ATP levels and the OD_600_ growth curve shown in Figure 1B, but this was not seen for bacteria exposed to DFMO. As ATP levels can be used as a surrogate measure of cell numbers—for comparably grown cultures—we presumed that the observed increase simply indicated continued growth of *H. pylori*. To eliminate this uncertainty we used the optical density of the cultures, and the equation derived from Figure 5D, to calculate the ATP-dependent luminescence per 10^6^ bacteria. This transformation demonstrated that the effect of DFMO was even greater than initially perceived. When compared to control cultures, DFMO induced a 1.5-fold increase in luminescence by 1 h that grew to 3.4-fold by 12 h (*p<*0.001; Figure 6B). We also observed that while the control culture maintained a relatively steady level of ATP/10^6^ bacteria throughout the entire time course (405.8±28.8 luminescence units), large dynamic changes were seen in the 1% DFMO cultures, suggesting that the chemical drastically altered the metabolism of *H. pylori*.

### DFMO reduces the proinflammatory effect of *H. pylori*

**Figure 7:**
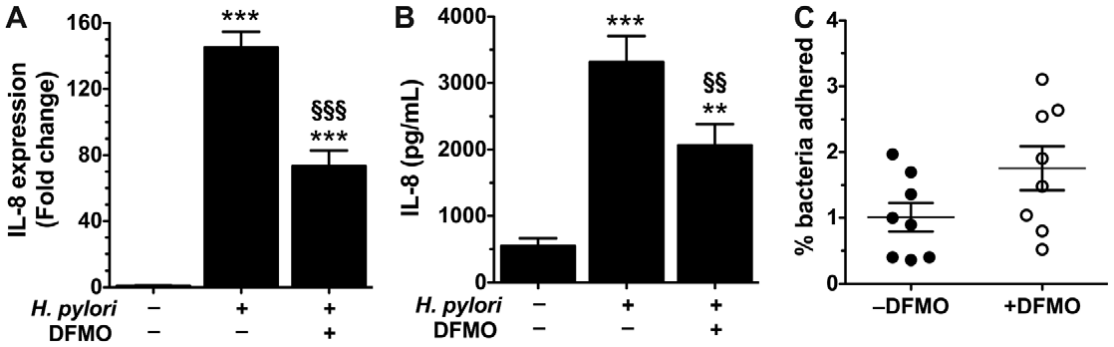
DFMO-treated *H. pylori* 60190 induce less IL-8 expression. Human gastric AGS cells were exposed to strain 60190 previously grown for 12 h with or without 1% DFMO and IL-8 expression was determined. (A) Real-time PCR was used to measure levels of IL-8 mRNA after 4 h of exposure. Bars indicate the mean level of gene expression relative to uninfected control cells (*n = *3). (B) IL-8 protein levels in supernatants were measured by ELISA after 6 h of infection. Bars indicate the mean IL-8 concentration (*n = *6). (C) After 2 h of infection adherent bacteria were quantified by serial dilution and plating. Each point is a single well of AGS cells and the means and standard errors are depicted (*n = *8). **, *p*<0.0; ***, *p<*0.001 *versus* the control condition; §§, *p<*0.01; §§§, *p<*0.001 *versus* cells infected with untreated bacteria.

Acute active inflammation characterized by neutrophil infiltration is a central constituent of the immune response to *H. pylori* infection. Chemokines, which are secreted chemoattractant proteins, are responsible for the influx of these cells to the site of infection. IL-8 is one such chemokine, and its expression has been reported to be increased in the gastric mucosa of infected patients [16], [17], [40] and in gastric epithelial cells inoculated with *H. pylori in vitro* [41], [42]. We cocultured human gastric epithelial AGS cells with *H. pylori* 60190, a strain known to induce IL-8 in these cells [43] at an MOI of 200 for 4 h and then analyzed mRNA levels (Figure 7A). *H. pylori* 60190 induced a 145.1±9.5-fold increase in IL-8 mRNA expression compared with untreated cells (*p<*0.001) and this induction was nearly halved when bacteria cultured in the presence of 1% DFMO for 12 h prior to addition to host cells were used (73.4±9.4-fold, *p<*0.001). We also measured IL-8 protein levels in supernatants of AGS cells cocultured with *H. pylori* 60190 at an MOI of 100 for 6 h (Figure 7B). Cells infected with bacteria grown without DFMO produced 3316±392 pg/mL IL-8, a 7.2±2.5-fold increase over control cells (*p<*0.001). When *H. pylori* cultured in the presence of 1% DFMO were used, AGS cells generated only 2066±316 pg/mL IL-8, a 47.8±6.7% inhibition (*p<*0.01). These data suggest that the immunostimulatory effect of the bacteria is lessened by exposure to DFMO.

Some studies have reported that coccoid-form *H. pylori* exhibit reduced adherence compared to helical-shaped bacteria and that this correlates with lesser induction of IL-8 [44], [45]. To determine if a similar effect occurred with the bacteria altered by DFMO, we compared adherence to AGS cells of *H. pylori* grown with or without 1% DFMO for 12 h. There was not a decrease, but rather there was a modest increase in adherence, with 1.01±0.22% of untreated bacteria and 1.75±0.33% of DFMO-treated bacteria found to be adherent, respectively (Figure 7C). Thus, the differential induction of IL-8 was likely due to induced changes in the bacteria and not simply disparities in adherence.

### CagA expression and translocation are affected by DFMO

**Figure 8:**
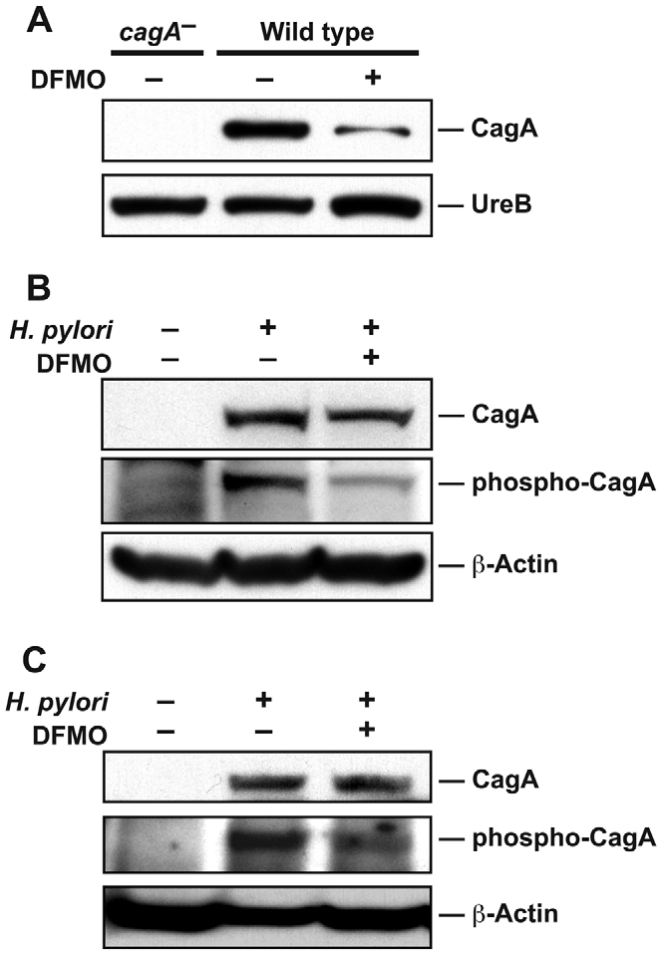
DFMO suppresses *H. pylori* CagA expression and its translocation into gastric epithelial cells. *H. pylori* strain 60190 was grown for 12 h with or without DFMO supplementation. (A) Western blot analysis of CagA and UreB expression in bacteria. An isogenic 60190 *cagA* mutant was used as a control. (B) Western blot analysis of total CagA, tyrosine-phosphorylated CagA, and β-actin in lysates prepared from AGS cells infected for 6 h with these bacteria or uninfected as a control. (C) Western blot analysis of total CagA, tyrosine-phosphorylated CagA, and β-actin in lysates prepared from AGS cells infected for 12 h or controls.

*H. pylori* possesses a number of virulence factors known to be important for the induction of disease during infection. One of the best described is the effector molecule CagA, which has been implicated in the disruption of tight junctions and alteration of cellular morphology [46]. As infection with CagA-expressing strains of *H. pylori* is associated with worse clinical outcome in patients, namely higher rates of peptic ulcer disease and gastric cancer [46], [47], [48], [49], we sought to determine if DFMO treatment could affect the production and function of this bacterial protein. We utilized *H. pylori* 60190 in this set of experiments, since it has been reported that SS1 has an incomplete *cag* pathogenicity island (*cag*-PAI) [50]. Bacteria cultured for 12 h in the presence of 1% DFMO exhibited lower levels of CagA protein expression compared to untreated cells (Figure 8A). This effect was not due to a global suppression of protein translation, as levels of UreB, a component of the highly-expressed bacterial urease, were not affected. Although we observed a decrease in CagA protein, mRNA levels were not affected by DFMO (Figure S3A), indicating that it acts in a post-transcriptional manner. This type of control of expression, which can involve altered mRNA stability, has been reported for other *H. pylori* genes [51], [52], [53]. We measured the stability of *cagA* from cultures grown with or without DFMO, but did not observe any increase in decay rate; in fact, we found that DFMO improved transcript stability and increased its half-life from 3.4 min to 4.3 min (Figure S3B).

CagA translocation is required in order for the protein to exert its effects on host cells [18], and once within the host cytoplasm CagA can be phosphorylated by tyrosine kinases [54], [55]. Figure 8B demonstrates that lysates of AGS cells infected with *H. pylori* grown with 1% DFMO prior to coculture with the host cells for 6 h contained lower levels of CagA protein and phosphorylated CagA (identified as a tyrosine-phosphorylated protein of ∼130 kDa) [56] than lysates of cells infected with control bacteria. When assessed after 12 h of coculture with AGS cells, the levels of CagA protein in lysates from AGS cells infected with *H. pylori* grown with DFMO had recovered, but the levels of phosphorylated CagA had not (Figure 8C). Differences in levels of phosphorylated CagA could indicate disruption of the T4SS responsible for its translocation. However, when we measured mRNA levels of *cagE* and *cagM*, two members of the *cag*-PAI that are essential components of the T4SS, exposure to DFMO for either 6 h or 12 h had no effect on the levels of these transcripts (Figures S3C and S3D). Taken together, these data indicate that exposure to DFMO affects the ability of *H. pylori* to produce and translocate CagA, a major virulence factor, adding to its potential as an antibacterial agent.

## Discussion

*H. pylori* is a major cause of mortality and morbidity worldwide. It is estimated that more than 50% of the human population is infected, with higher prevalence in the developing world and less in industrialized countries [57], [58]. Most infections induce only mild gastritis, but if left untreated they can lead to gastric and duodenal ulceration or gastric cancer. Standard treatment regimens are known to be far less than 100% effective [3], [4], [5], [59], indicating that novel therapies could be useful, especially given high rates of antibiotic resistance worldwide [59]. In our recent publication on the protective effect of DFMO administration on *H. pylori*-induced gastritis in mice—indicated by reduced inflammation and colonization—we concluded that it was attributable to the beneficial effects of the chemical's inhibition of host ODC activity [6]. In the current report we have demonstrated that a direct effect of DFMO on the bacteria may also be important.

Our data indicate that exposure to 1% DFMO suppresses growth of *H. pylori* such that generation time is nearly doubled. The mechanism of action does not appear to involve polyamine synthesis since there was no detectable ornithine decarboxylase activity in the bacteria and supplementation with downstream polyamine products was unable to reverse the effect. In addition, other Gram-negative bacterial species that do contain ODC were found to be insensitive to the inhibitory effect of DFMO. This lack of effect on other bacteria suggests that the DFMO is not overtly toxic at this dose. Additionally, the concept that the action of DFMO was not a result of direct toxicity of the chemical was supported by our finding that *H. pylori* were not immediately killed, and in fact responded with increased ATP levels. We found that the induced alterations required continuous exposure to DFMO and were reversible by halting exposure. We also noted that the alterations to growth rate and metabolism could be observed as a change in bacterial morphology, with DFMO causing the *H. pylori* to adopt a more coccoid form. These altered bacteria expressed lower levels of CagA, translocated less CagA, and induced 50% less IL-8 expression in gastric epithelial cells than untreated bacteria, despite there being no difference in adherence to these epithelial cells *in vitro*.

In order to affect growth of *H. pylori* a high concentration of 1% DFMO (equivalent to 55 mM) was required. This dose was the same as was administered to mice *ad libitum* in our previous study [6]. Given that a mouse weighs between 20 and 25 g and consumes 10–15 mL of water daily, this implies that each mouse receives a daily DFMO dosage in the range of 4000–7500 mg/kg. Allometric dose translation [60] for human administration would be approximately 500 mg/kg. Although this dose is high, similar or higher levels have been used for treatment of trypanosomiasis, via inhibition of the parasite's ODC. Clarkson et al. [61] cured experimentally infected mice with a 14-day course of 2% DFMO and a single dose of the anti-protozoal agent suramin. The same group also reported that late-stage human sleeping sickness could be cured by 14 days of intravenous 400 mg/kg DFMO followed by 3–4 weeks of oral dosage at 300 mg/kg [62]. Although there were side effects at this dosage, including diarrhea and abdominal pain, all were reversible, and treatment of *H. pylori* would likely not require such a lengthy period of treatment.

Previous studies have indicated that exposure of *E. coli* or *Pseudomonas aeruginosa* to the polyamine synthesis inhibitors monofluoromethylornithine, difluoromethylarginine, and dicyclohexylammonium sulfate significantly increased bacterial generation times and could be overcome by addition of exogenous polyamines [63], [64]. However, the inhibitory effect on *H. pylori* growth likely involves an alternative mechanism, as the genome of this species contains no recognized ODC gene, and the bacteria possess no ODC activity. In our studies we also found that *E. coli* and *C. rodentium*, two bacterial species that do possess an ODC, were completely insensitive to the inhibitory effects of DFMO. The polyamine pathways of *H. pylori* are not completely understood and may be unique, as the species is missing a number of biosynthetic enzymes found in other bacteria [12], [13], [14], [15]. In addition, the sequenced genomes of *H. pylori* contain no recognizable polyamine transporters. It has been suggested that in the high-acid environment of the stomach all polyamines would be completely protonated and therefore unable to be acted on by known polyamine binding proteins [11]. In our experiments, DFMO had no effect on intracellular or extracellular polyamine levels of bacterial cultures and supplementation with either putrescine or spermidine did not restore the growth rate to normal levels. Although none of our findings indicate the involvement of polyamine metabolism in the mechanism of action of DFMO on *H. pylori*, others have targeted polyamine synthesis in this bacterium using methylglyoxal bis(cyclopentylamidinohydrazone) (MGBCP), an inhibitor of multiple enzymes, including spermidine synthase [65]. Exposure to MGBCP caused a dramatic reduction in the growth of *H. pylori in vitro*, but the authors suggested that the localization of *H. pylori* within the mucus layer of the highly acidic stomach could limit its chemotherapeutic effectiveness. Our previous study [6] indicates that DFMO is effective *in vivo* in a mouse model when administered continuously after inoculation with *H. pylori*.

In our recently published report we noted that treatment of *H. pylori*-infected mice with DFMO led to decreased colonization via enhanced antimicrobial nitric oxide generation by host macrophages, which we attributed to the drug's effect on host cell ODC that can otherwise inhibit translation of inducible nitric oxide synthase [6]. The current study suggests that direct effects of DFMO on the bacteria may play a role as well. When we attempted to separate these two effects by infecting mice with *H. pylori* SS1 pre-treated with DFMO for 12 h, after 1 month of infection without treatment of the mice with DFMO, there was no observable difference in colonization. We attributed this to the rapid recovery of the bacteria once they were in the mouse stomach and no longer exposed to DFMO, a supposition that was supported when we performed growth curves in which we removed DFMO supplementation from growing bacterial cultures and observed the swift improvement of growth rate to normal levels. In the future we will determine the relative importance of the drug's effects on the bacteria and host by administering DFMO to heterozygous ODC^+/−^ mice [66], [67], which we will use because homozygous deletion of ODC is lethal [8]. If the reduced gastritis and colonization are recapitulated in infected ODC^+/−^ mice, and if there is an even greater reduction with DFMO treatment, this could suggest that drug effects on the bacteria are important for its ameliorative properties.

*H. pylori* morphologic form may have a bearing on to its viability and infectivity, but the literature on *H. pylori* coccoid forms is not consistent. Coccoid forms were observed in one prospective study of human gastric biopsies in 83% of cases [68] and it has been suggested that they may provide a source for the spread of infection or of recrudescence in patients reportedly cured [69]. Other reports on coccoid forms have reached a variety of conclusions: that coccoid bacteria are a manifestation of cell death [70]; that they are a viable, but non-culturable form [71]; that coccoid forms can successfully infect mice [72], [73]; and that they are unable to colonize neonatal pigs [74]. Irrespective of the controversy surrounding the nature of coccoid bacteria, the morphologically altered *H. pylori* we observed may differ from those seen in other studies. The changes we report here occur very rapidly, after only 6 h of exposure to DFMO, while other studies induced coccoid forms by multi-day culture or exposure to antibiotics [72]. In addition, whereas some have noted a decrease in cellular activity following conversion of *H. pylori* to a coccoid form [70], we measured higher internal levels of ATP after DFMO treatment. This observed increase could indicate an upregulation of ATP production or a downregulation of ATP usage leading to an accumulation of the molecule. The latter seems more likely as it correlates with the decrease in growth rate that we demonstrated. Another recent discovery is that the state of peptidoglycan crosslinking in *H. pylori* plays an important role in maintaining its helical shape that is important in its motility and infectivity [75]. While knockouts of cell shape determinant (*csd*) genes did not affect growth *in vitro* in that study, it still could prove important to investigate the effects of DFMO on peptidoglycan and associated *csd* genes in future experiments.

Increased expression of IL-8 is part of the immune response to *H. pylori* infection *in vivo* [40]. As the known interaction of DFMO with host cells prevents discrimination of the effect of DFMO on bacteria in the stomach we performed *in vitro* experiments using the human gastric AGS cell line in the absence of DFMO in the cocultures and observed that IL-8 expression was significantly reduced. Some studies using coccoid-form bacteria have reported that they induce lower levels of IL-8 in epithelial cells, and, concomitantly, adhere to a lesser degree than helical *H. pylori* [44], [45]. Our data indicate no loss of adherence of DFMO-treated bacteria, suggesting that other alterations of bacterial physiology are responsible for the reduction in IL-8 expression. Production of the chemokine IL-8 by the gastric epithelium elicits an influx of neutrophils that are the hallmark of the acute gastritis associated with *H. pylori* infection [46], [47], [76]. Thus, administration of DFMO to patients could lead to reduced IL-8 expression by epithelial cells and a reduction of inflammation.

Urease is an important virulence factor of *H. pylori*. It is responsible for the hydrolysis of urea into ammonia that acts to rapidly neutralize the low pH of the acidic stomach environment. We utilized UreB, a subunit of the urease enzyme, as a control in our bacterial Western blots and noted no decrease in its expression with DFMO exposure. To further investigate if urease was affected by DFMO, we measured enzymatic activity in both *H. pylori* SS1 and 60190. Consistent with the Western blot results, DFMO had no effect on the urease activity after either 3 or 6 h of culture (Figure S5).

The *cag*-PAI comprises a set of virulence genes encoding the components of a T4SS able to deliver bacterial products into host cell cytoplasm. The bacterial effector protein CagA is encoded within the *cag*-PAI and when introduced into epithelial cells it triggers a variety of signaling events that affect host cell processes [46], [47]. Some reports have indicated that IL-8 production by AGS cells is CagA-dependent [77], [78], though it should be recognized that other studies have reported a CagA-independent induction of this chemokine [43], [79]. We observed that bacteria grown in media containing 1% DFMO exhibited decreased CagA protein expression and that AGS cells incubated with such bacteria contained lower levels of phosphorylated CagA, indicating an additional potential benefit for the chemotherapeutic use of this drug. Interestingly, prolonged coculture of *H. pylori* with AGS cells led to recovery from the DFMO-induced suppression of total CagA protein levels so that after 12 h no difference remained. Since the phosphorylated form of CagA is found only inside AGS cells, while total CagA levels are indicative of both the translocated protein and that remaining in the bacteria adherent to the epithelial cells, our data indicate that the effect of DFMO on CagA expression dissipates more rapidly during the recovery of *H. pylori* in AGS medium than does its effect on phospho-CagA levels. The persistent inhibition of CagA phosphorylation may be due to inhibition of translocation of CagA via post-transcriptional effects on genes involved in the T4SS. In addition, we have found that AGS cell culture medium poorly supports *H. pylori* growth, and it is much more restrictive than Brucella broth (Figure S4), and thus it is possible that the apparent normalization of CagA levels are part of an overall stress response that overshadows the effect of DFMO. Our data suggest that DFMO alters CagA expression via a post-transcriptional mechanism. Such control has been reported for other *H. pylori* genes including the flagellar protein FlaA [51], neutrophil activating protein (Nap) A [52], and the global regulator HP1043 [53]. FlaA protein levels are partially regulated by destabilization of the *flaA* mRNA transcript [51], but when we measured stability of *cagA* from cultures grown with or without DFMO there was no increase in decay rate that could account for the decreased level of CagA protein. It should be noted that while some human patients are infected with *cagA* ^–^ strains [46], [47], [48], [49], DFMO is likely to still have beneficial effects in such people, because of its growth inhibitory effects. It is also likely that other effects on *H. pylori* genes could be occurring, which could be addressed by discovery studies using proteomic or microarray analyses.

Although we have not determined the molecular mechanism by which DFMO suppresses *H. pylori* growth, this drug may be a useful adjunctive treatment for human *H. pylori* infections. DFMO is used as treatment for trypanosomiasis, as mentioned above, and oral DFMO (500 mg daily) has been found safe and effective as part of a chemopreventive regimen for recurrent colorectal adenomas [80]. To our knowledge, there are no reports on the usage of DFMO as an antibacterial agent *in vivo*, despite the fact that interference with the polyamine biosynthetic pathways could be an attractive target for retarding fast-growing infections. Although we would not suggest its use as a monotherapy, DFMO treatment could be considered as a future addition to current standard regimens, especially in regions of the world where *H. pylori* exhibits high levels of antibiotic resistance.

## Supporting Information

Figure S1 **Polyamine levels in growth medium are unaffected by DFMO.** *H. pylori* SS1 were grown with or without 1% DFMO and growth medium samples were taken over 12 h. Putrescine (A), spermidine (B), and spermine (C) levels were determined by HPLC. The dashed line in each panel denotes the polyamine level measured in uninoculated broth. Each point represents the mean and standard error (*n = *3).
(TIF)

Figure S2 **Polyamine levels in** ***H. pylori*** **are unaffected by DFMO.** *H. pylori* SS1 were grown with or without 1% DFMO and bacteria were sampled over 12 h. HPLC was used to measure spermidine (A) and spermine (B) levels in bacterial lysates (1×10^7^ *H. pylori* in 400 µL buffer). Each point represents the mean and standard error (*n = *3).
(TIF)

Figure S3 **DFMO does not affect** ***cag*** **transcript levels or** ***cagA*** **mRNA stability.** *H. pylori* 60190 were grown with or without 1% DFMO for 6 or 12 h. mRNA levels of *cagA* (A), *cagE* (C), or *cagM* (D) were determined by real-time PCR. Bars indicate the mean level of gene expression relative to uninfected control cells at each time point (*n = *3). (B) *cagA* mRNA stability was determined by adding the transcription inhibitor rifampicin to *H. pylori* cultures grown for 6 h. Transcript levels were quantified at 15 min intervals by real-time PCR and transcript half-lives (λ) were calculated by plotting an exponential decay curve to each data set. Each point represents the mean and standard error of the remaining *cagA* transcript compared to the 0 min time point as a percentage (*n = *3).
(TIF)

Figure S4 ***H. pylori*** **growth is stunted in AGS cell medium.** Cultures of F12 medium, some containing 1% (w/v) DFMO, were inoculated with *H. pylori* SS1 at an OD_600_ of ∼0.1 and growth was monitored for 24 h by measuring OD_600_ at the indicated time points. Solid lines depict the growth curve obtained for each treatment and error bars represent the standard error (*n = *3), while the dashed lines indicate the calculated exponential regression curves using the first 12 h of data. The generation time (g) and goodness of fit (R^2^) are indicated for each curve.
(TIF)

Figure S5 **DFMO does not affect urease activity in** ***H. pylori*** **.** *H. pylori* SS1 (A) and 60190 (B) were grown with or without 1% DFMO and bacteria were sampled at 3 or 6 h. *H. pylori* were flash frozen in liquid nitrogen then 1×10^6^ were incubated for 60 min in a detection solution containing urea and phenol red. Activity was determined from the slope of a best-fit line on a plot of OD_550_ versus time. Bars indicate the mean urease activity relative to the control cells at each time point (*n* = 3).
(TIF)
