# Supplementary figures and images for: Difluoromethylornithine Is a Novel Inhibitor of Helicobacter pylori Growth, CagA Translocation, and Interleukin-8 Induction
Source: PLoS One. 2011 Feb 28;6(2):e17510. doi: 10.1371/journal.pone.0017510 (PMC3046249; doi:10.1371/journal.pone.0017510)

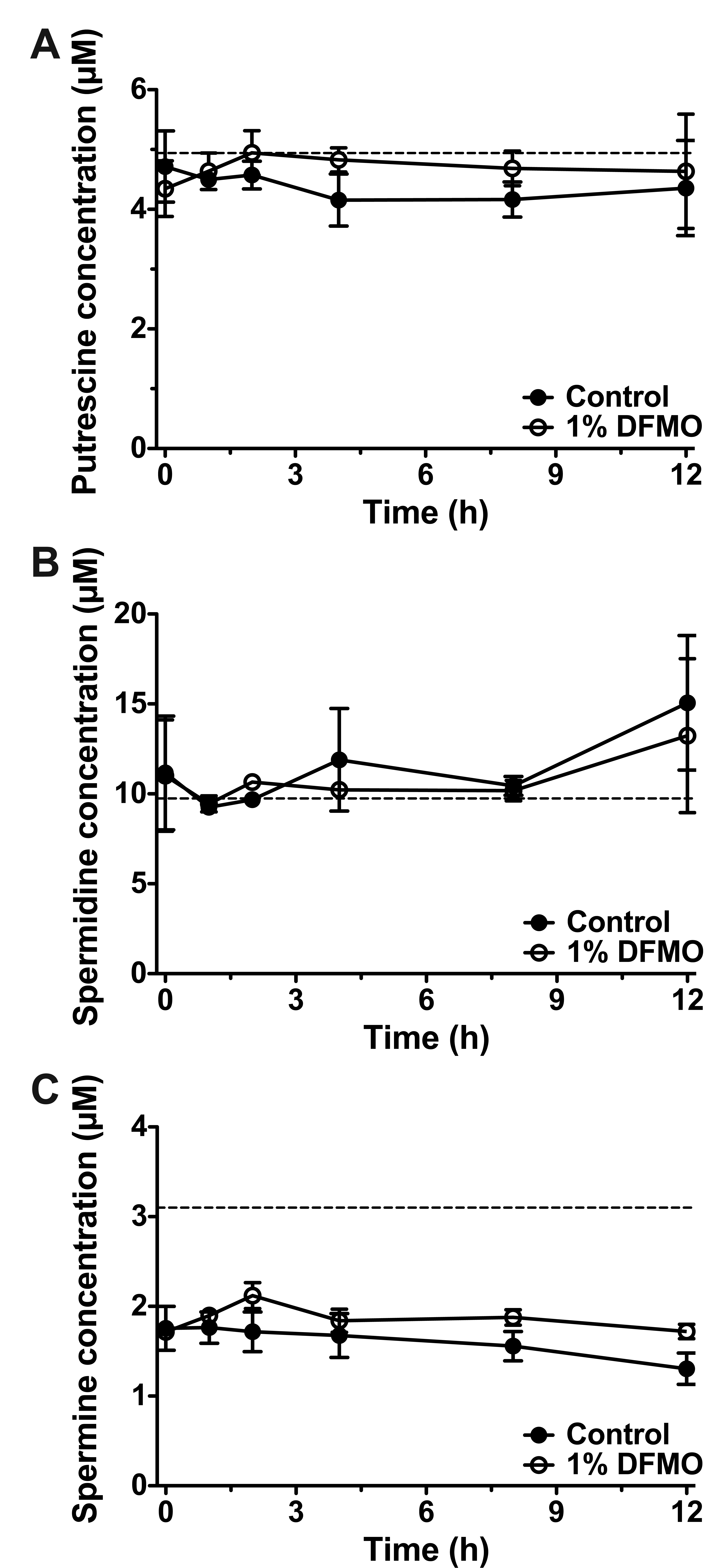

Supplement: Figure S1 — Polyamine levels in growth medium are unaffected by DFMO. H. pylori SS1 were grown with or without 1% DFMO and growth medium samples were taken over 12 h. Putrescine (A), spermidine (B), and spermine (C) levels were determined by HPLC. The dashed line in each panel denotes the polyamine level measured in uninoculated broth. Each point represents the mean and standard error (n = 3). (TIF) [file pone.0017510.s001.tif]

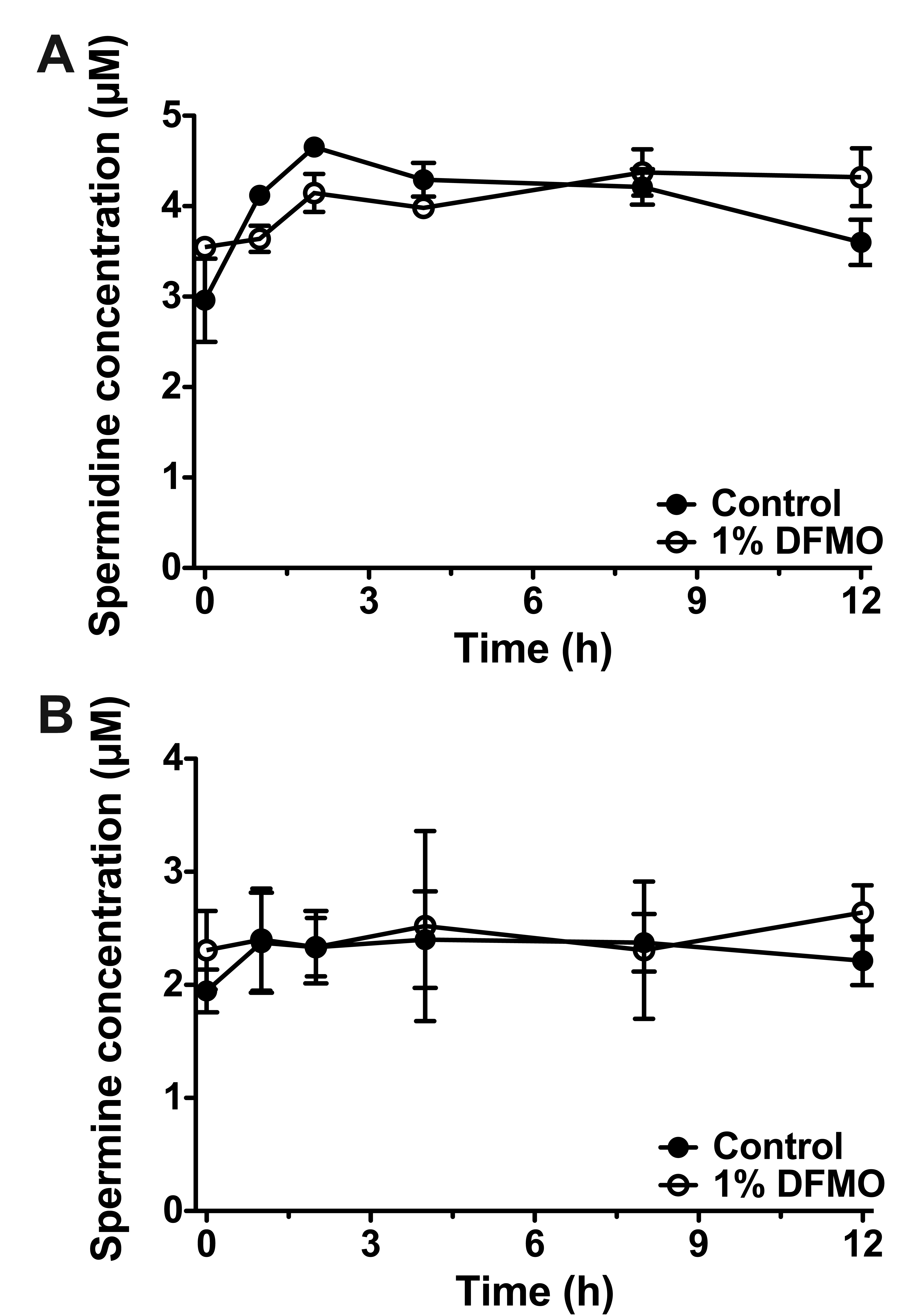

Supplement: Figure S2 — Polyamine levels in H. pylori are unaffected by DFMO. H. pylori SS1 were grown with or without 1% DFMO and bacteria were sampled over 12 h. HPLC was used to measure spermidine (A) and spermine (B) levels in bacterial lysates (1×10^7^ H. pylori in 400 µL buffer). Each point represents the mean and standard error (n = 3). (TIF) [file pone.0017510.s002.tif]

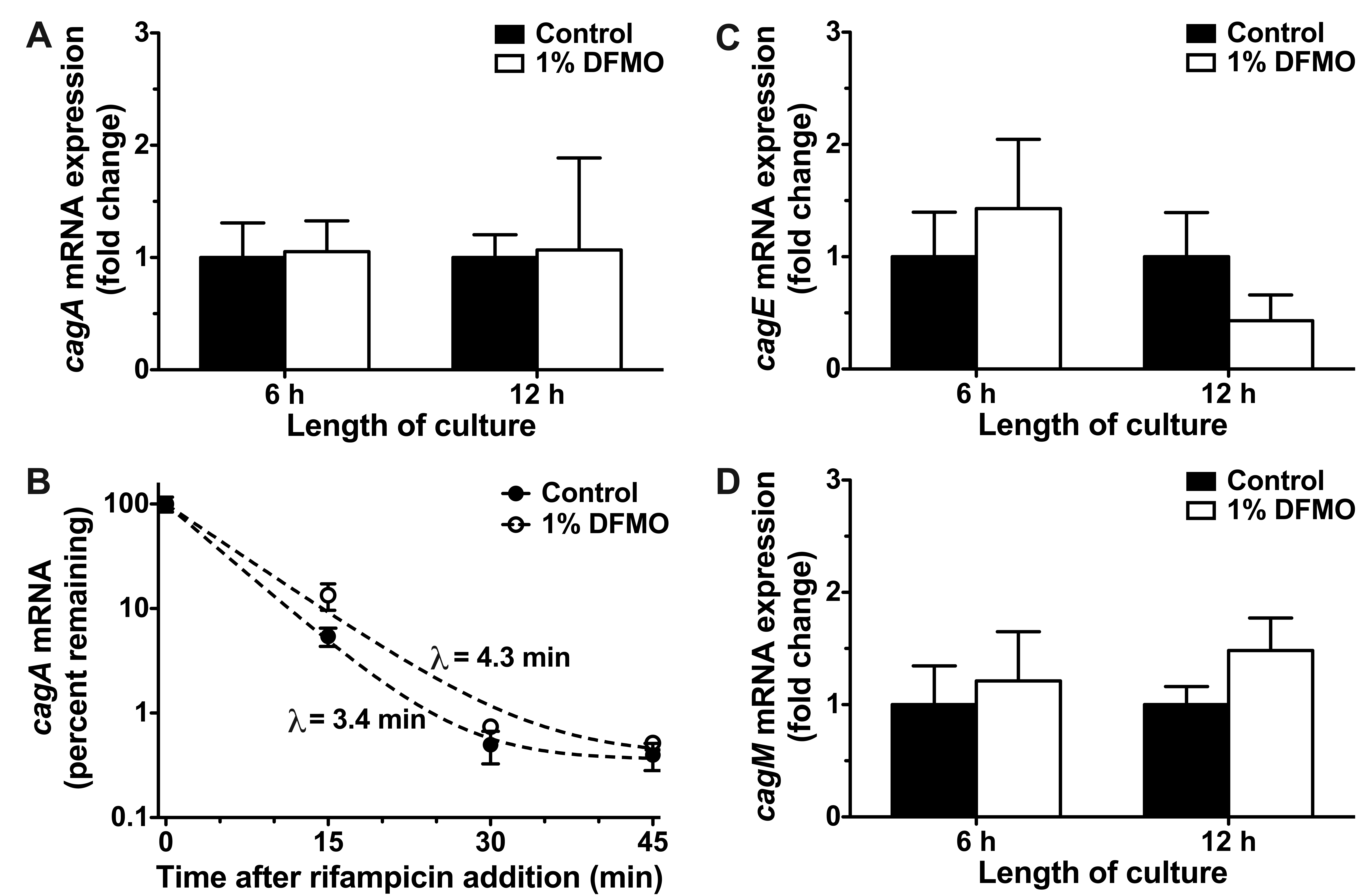

Supplement: Figure S3 — DFMO does not affect cag transcript levels or cagA mRNA stability. H. pylori 60190 were grown with or without 1% DFMO for 6 or 12 h. mRNA levels of cagA (A), cagE (C), or cagM (D) were determined by real-time PCR. Bars indicate the mean level of gene expression relative to uninfected control cells at each time point (n = 3). (B) cagA mRNA stability was determined by adding the transcription inhibitor rifampicin to H. pylori cultures grown for 6 h. Transcript levels were quantified at 15 min intervals by real-time PCR and transcript half-lives (λ) were calculated by plotting an exponential decay curve to each data set. Each point represents the mean and standard error of the remaining cagA transcript compared to the 0 min time point as a percentage (n = 3). (TIF) [file pone.0017510.s003.tif]

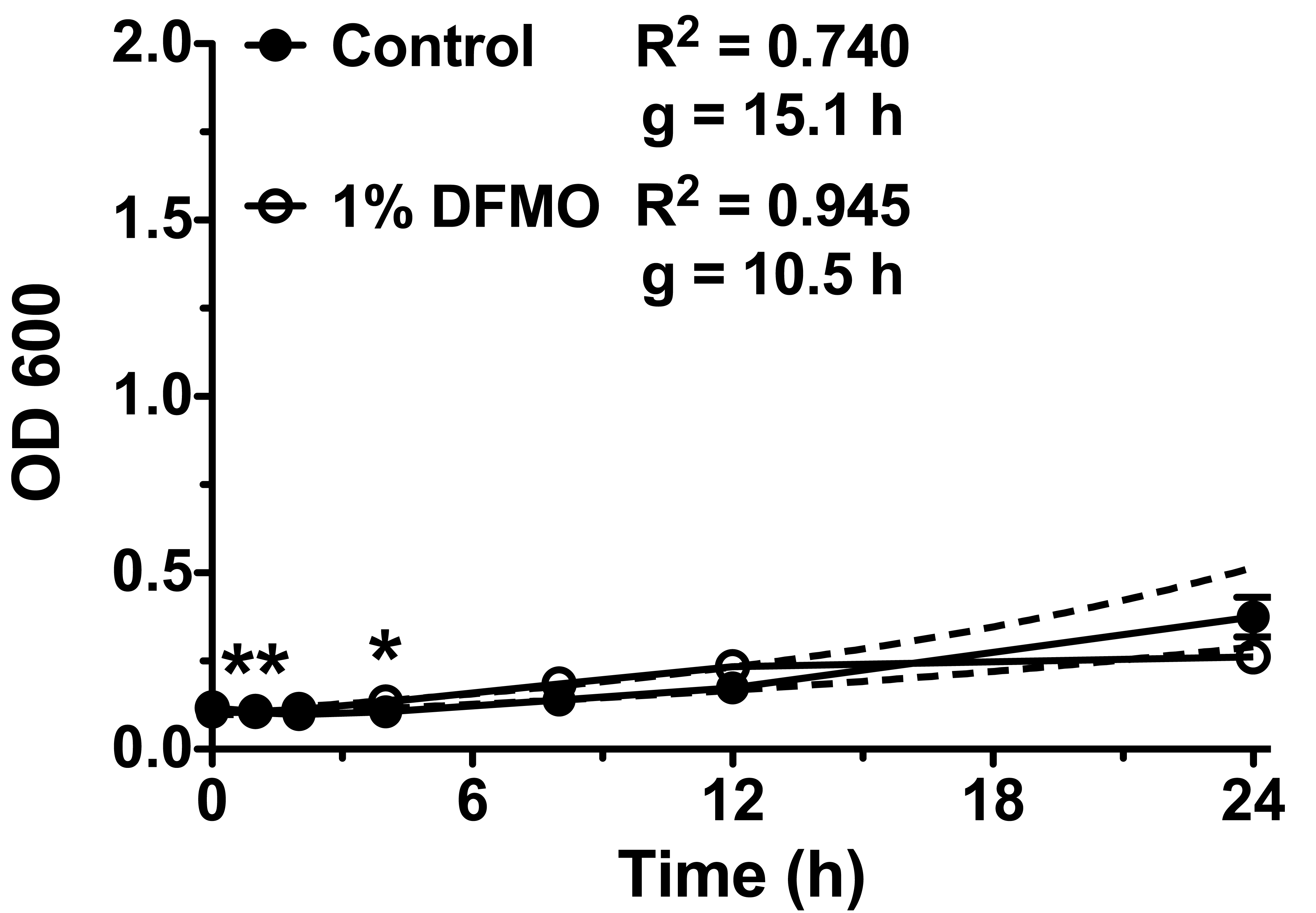

Supplement: Figure S4 — H. pylori growth is stunted in AGS cell medium. Cultures of F12 medium, some containing 1% (w/v) DFMO, were inoculated with H. pylori SS1 at an OD600 of ∼0.1 and growth was monitored for 24 h by measuring OD600 at the indicated time points. Solid lines depict the growth curve obtained for each treatment and error bars represent the standard error (n = 3), while the dashed lines indicate the calculated exponential regression curves using the first 12 h of data. The generation time (g) and goodness of fit (R2) are indicated for each curve. (TIF) [file pone.0017510.s004.tif]

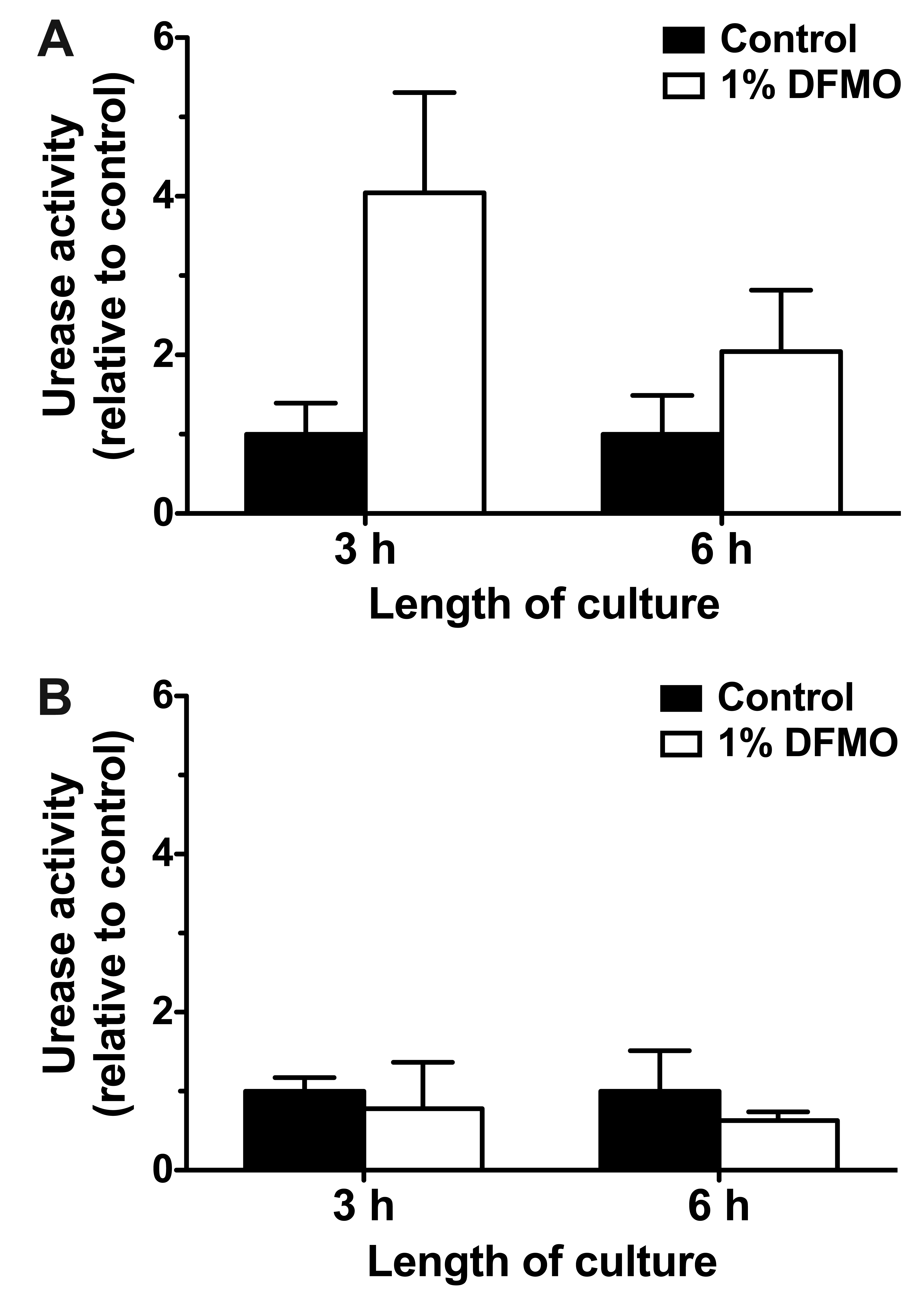

Supplement: Figure S5 — DFMO does not affect urease activity in H. pylori . H. pylori SS1 (A) and 60190 (B) were grown with or without 1% DFMO and bacteria were sampled at 3 or 6 h. H. pylori were flash frozen in liquid nitrogen then 1×10^6^ were incubated for 60 min in a detection solution containing urea and phenol red. Activity was determined from the slope of a best-fit line on a plot of OD550 versus time. Bars indicate the mean urease activity relative to the control cells at each time point (n = 3). (TIF) [file pone.0017510.s005.tif]
